# Thermal Preference of Juvenile Dover Sole (*Solea solea*) in Relation to Thermal Acclimation and Optimal Growth Temperature

**DOI:** 10.1371/journal.pone.0061357

**Published:** 2013-04-22

**Authors:** Edward Schram, Stijn Bierman, Lorna R. Teal, Olga Haenen, Hans van de Vis, Adriaan D. Rijnsdorp

**Affiliations:** 1 IMARES, Wageningen UR, IJmuiden, The Netherlands; 2 Central Veterinary Institute, Wageningen UR, Lelystad, The Netherlands; 3 Aquaculture and Fisheries Group, Wageningen UR, Wageningen, The Netherlands; University of Sao Paulo, Brazil

## Abstract

Dover sole (*Solea solea*) is an obligate ectotherm with a natural thermal habitat ranging from approximately 5 to 27°C. Thermal optima for growth lie in the range of 20 to 25°C. More precise information on thermal optima for growth is needed for cost-effective Dover sole aquaculture. The main objective of this study was to determine the optimal growth temperature of juvenile Dover sole (*Solea solea*) and in addition to test the hypothesis that the *final preferendum* equals the optimal growth temperature. Temperature preference was measured in a circular preference chamber for Dover sole acclimated to 18, 22 and 28°C. Optimal growth temperature was measured by rearing Dover sole at 19, 22, 25 and 28°C. The optimal growth temperature resulting from this growth experiment was 22.7°C for Dover sole with a size between 30 to 50 g. The temperature preferred by juvenile Dover sole increases with acclimation temperature and exceeds the optimal temperature for growth. A *final preferendum* could not be detected. Although a confounding effect of behavioural fever on temperature preference could not be entirely excluded, thermal preference and thermal optima for physiological processes seem to be unrelated in Dover sole.

## Introduction

Fish are obligate ectotherms and most, if not all, physiological processes in fish are under the influence of body temperature [1]. Body temperature is essentially the same as ambient water temperature in fish of less than 1 kg body weight (Reynolds et al 1976 in [2]). Maximum performance of physiological processes occurs at species specific thermal optima. Thermal optima in fish have been found to coincide with thermal preferences [3] and this thought to be the result of coadaptation by natural selection [4]. It allows fish to optimize the performance of physiological processes in a heterogeneous thermal environment by thermoregulatory behaviour. Thermoregulatory behaviour is affected by complex trade-offs between internal processes with different thermal optima as well as by external factors such as food availability and predation risk [4]. Thermoregulatory behaviour of fish may also be affected by their thermal history. When exposed to a temperature gradient the thermal preference of fish may initially be under the influence of their previous thermal acclimation (acute preference). Acute preference then increases with acclimation temperature until they are equal. The temperature at which this occurs was defined as the *final preferendum* by Fry in 1947 [3]. Prolonged exposure to a temperature gradient ultimately results in fish gravitating to this *final preferendum*. Acute thermal preference close to the *final preferendum* independent from thermal acclimation has also been reported in fish [5].

Thermal biology and physiology of fish is of great interest to the more applied research field of aquaculture. The aquaculture industry aims to produce aquatic organisms under controlled conditions in a cost-effective manner. Water temperatures in aquaculture production systems should obviously lie within critical upper and lower thermal limits of the cultured organisms. In addition, water temperatures are preferably kept close to species specific optimal growth temperatures as cost-effective aquaculture in most cases demands a high biomass gain per unit of effort. Optimal growth temperatures have consequently been investigated in growth experiments for many fish species of commercial interest to the aquaculture industry, for example turbot [6], Atlantic cod [7], Atlantic salmon and rainbow trout [8]. Jobling (1981) proposed to determine optimal growth temperatures by using the concept of *final preferendum* in temperature preference tests as a practical alternative for long-term growth experiments [9].

Dover sole (*Solea solea*, order *Pleuronectiformes*, family *Soleidae*, Linnaeus, 1758) was first recognized as a high potential species for commercial aquaculture some thirty years ago [10]. Various aspects of Dover sole biology relevant to its aquaculture were extensively studied over the last three decades, see Imsland et al (2003) [11] for a review, including important but limited work on optimal growth temperatures [12],[13]. Irvin (1973) [13] showed that growth of juveniles (TL ca. 5 cm) increased with temperature up to 23°C and then dropped at 27°C. Slightly larger juveniles (TL 12–13 cm) showed increasing growth with temperatures up to 25°C [12]. In both studies little increase in growth was observed above 20°C. Based on these results it was concluded that the optimal growth temperature lies between 20 and 25°C [12]. This temperature range for optimal growth is rather imprecise considering that maximising growth is particularly crucial for Dover sole aquaculture. Cost-effective production of Dover sole is hampered by low yields per unit of culture area due to relatively low growth and relatively low fish densities [14]. Clearly more detailed information on optimal growth temperatures is needed to ensure that in aquaculture Dover soles are reared at optimal temperatures.

The specific objective of our study was to obtain a more precise estimate of the optimal growth temperature of juvenile Dover sole. More generally we wanted to contribute to the understanding of the thermal biology of ectothermic vertebrates, in particular their thermal acclimation, by exploring the relations between acute thermal preference, thermal acclimation, thermal optima for growth and the *final preferendum*. We aimed to establish whether measurement of the *final preferendum* can be used to estimate the optimal growth temperature of fish as proposed by Jobling (1981) [9].

We investigated the optimal growth temperature of juvenile Dover sole by measuring the acute temperature preference in relation to acclimation temperature. We predicted that exposure of Dover sole to a temperature gradient would result in a behavioural response leading to a fish distribution along the temperature gradient representative of its thermal preference. Based on current insight in the optimal growth temperature [12], we predicted that the *final preferendum* of Dover sole lies in the range of 20 to 25°C. We also measured temperature preference after prolonged (24 h) exposure to a temperature gradient and predicted that the preferred temperature would gravitate to a *final preferendum* in the range of 20 to 25°C and be independent of the acclimation temperature. Finally we predicted that the *final preferendum* resulting from the temperature preference tests would equal the optimal growth temperature that we measured in a growth experiment.

## Materials and Methods

### Ethics statement and experimental animals

The treatment of the fish in this study was in accordance with Dutch law concerning animal welfare, as tested and approved of by the ethical committee for animal experimentation of Wageningen UR Livestock Research (number 2011046.b).

All Dover sole (*S. solea*) used in the preference tests and growth experiment were born and raised in captivity at Solea BV, IJmuiden, The Netherlands, as first generation (G1) progenies of wild brood stock collected in the southern North Sea. Brood stocks were kept in groups consisting of 15 males and 15 females. Each group was housed in a separate tank to allow the induction of natural spawning by temperature and photoperiod manipulation. Fertilized eggs where produced in spring (March–April) and autumn (September–October) by different groups. Individual batches of fertilized eggs produced by a single brood stock were kept separate during hatching and larval rearing. After weaning to a dry formulated diet, batches of juveniles originating from a single brood stock were merged into a single group for commercial ongrowing to market-sized fish. For the preference tests experimental animals with individual weights ranging from 20 to 30 g were collected randomly in December 2010 from a group of juveniles originating from batches of eggs produced in March–April 2010. For the growth experiment experimental animals with individual weights ranging from 30 to 40 g were collected randomly in September 2011 from a group of juvenile sole consisting of batches produced in September–October 2010.

### Temperature preference tests

#### Temperature preference chamber

**Figure 1:**
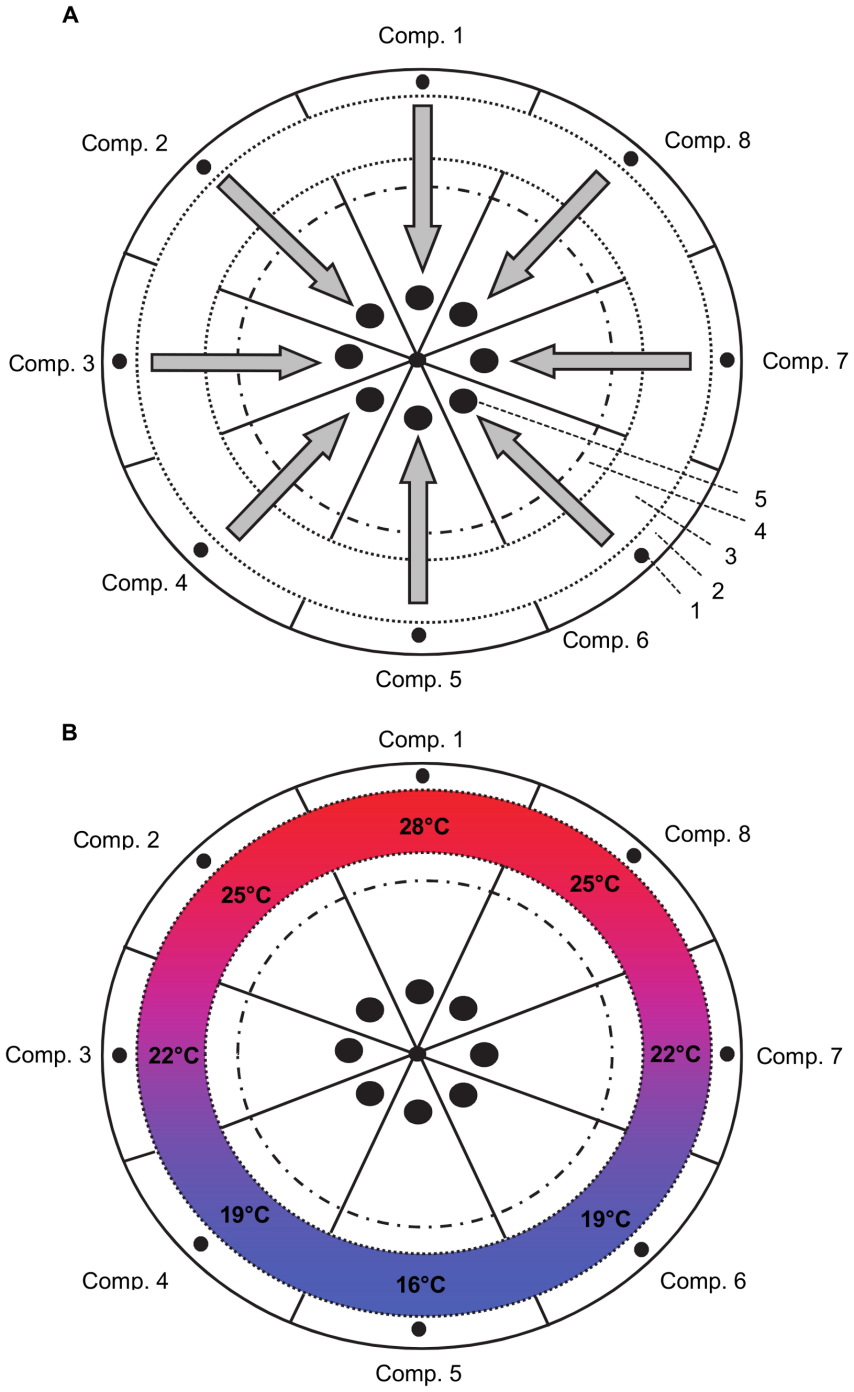
Schematic presentation of the preference chamber. Top view (A) presents schematically the water flows (arrows) and the main elements: 1) water inflow, 2) mixing channel, 3) swimming channel, 4) effluent channel, 5) central section with water drain. Top view (B) presents schematically the temperature gradient in the preference chamber.

To measure temperature preferences in fish a temperature preference chamber was built based on the design by Myrick et al (2004) [15]. The preference chamber was constructed from sand-coloured polypropylene and consisted of three rings and a middle section (Fig. 1A). Inflowing water was received in the outermost ring (*mixing channel*). The *mixing channel* was divided in eight compartments of equal size. Forty round holes per compartment (5 mm) in the inner wall of the *mixing channel* allowed for the water to flow to the second ring, the *swimming channel*. The *swimming channel* was not physically divided into compartments to allow for undisturbed movement of test fish. A high flow rate (∼10 L/min) limited mixing of water from adjacent flows in the *swimming channel* which resulted in the installation of a water temperature gradient in the *swimming channel*. The eight compartments in the *mixing channel* allowed for a gradient consisting of five different water temperature zones in the *swimming channel*: one compartment for the highest and lowest temperature zones, and two compartments for the three intermediate temperature zones (Fig. 1B). Forty round holes per compartment (5 mm) in the inner wall of the *swimming channel* allowed for the water to flow to the third ring, the *effluent channel*, which was again divided into eight equal compartments. From the *effluent channel* 20 round holes per compartment (10 mm) allowed the water to flow to the central section of the preference chamber. In the original design [15] all water flows reunite after passing the *effluent channel* in the central section of the preference chamber and are then discharged via a central drain. We modified the original design by introducing extra walls in the central section to keep water flows separate after the *effluent channel*. Each of the eight resulting pie-shaped compartments was equipped with a separate drain, enabling water recirculation over the preference chamber. Water flowing out of the preference chamber was collected in separate water storage tanks (60 L) for each temperature. From the water storage tanks water was pumped back into the *mixing channel*, closing the water recycling loop. The water storage tanks were equipped with thermostatically controlled heaters or coolers to create the desired water temperature. The preference chamber was filled with natural seawater. During the three day temperature preference test (see below) no water was exchanged. After each temperature test approximately 80% of the water in the preference chamber and water storage tanks was replaced by new natural seawater. Dimensions and settings of the preference chamber are presented in Table 1. Four video cameras connected to a recorder were positioned above the preference chamber to record the distribution of the fish over the preference chamber without disturbing the fish.

**Table 1:** Dimensions and settings of the preference chamber.

Parameter	Value
Total radius (cm)	95.0
Width *mixing channel* (cm)	9.5
Width *swimming channel* (cm)	19.0
Width *effluent channel* (cm)	9.5
Surface area *swimming channel* (m^2^)	0.91
Radius central section (cm)	57.0
Water height *swimming channel* (cm)	5.0
Total water volume *swimming channel* (L)	45.4
Total water volume preference chamber (L)	395
Average flow rate per section (L/min)	10
Average hydraulic residence time *swimming channel* (sec)	34
Average water velocity (cm/sec)	0.6

#### Pre-experimental thermal acclimation of fish

Dover sole (N = 288) with a mean (SD) weight of 23.9 (3.3) g, corresponding to a total length ranging between 12.5 and 14.5 cm, were randomly distributed over 18 aquaria (70×70 cm, 50 L, 16 fish per aquarium). Aquaria were part of a recirculating system including a 15.6 m^2^ sedimentation tank, a drum filter (Hydrotech) and a 3.4 m^3^ trickling filter (200 m^2^/m^3^ filter material) with a water temperature of 18.0°C. Each aquarium was equipped with a belt feeder to supply the daily feed ration of 1% of the mean fish weight per day. Feed was supplied over a period of 20 hours starting at 9:00. Fish were allowed to acclimate to these housing conditions for two weeks before the pre-experimental acclimation to three different water temperatures started.

Treatments consisted of pre-experimental acclimation of fish to 18, 22 or 28°C for 14 days prior to temperature preference tests in the preference chamber. For each treatment six aquaria (6×16 fish) were used. Mean individual fish weights were equal among treatments (ANOVA, p = 0.26). To heat the water to the desired temperature, aquaria were equipped with thermostatically controlled heaters (not required for acclimation to 18°C). Ambient water temperature of 18°C was increased by 2°C per day until the desired acclimation temperature was reached. The day the desired acclimation temperature was reached was the first day of the 14 day acclimation period. As the temperature preference tests took three days to complete for each group of fish (see below), pre-experimental temperature acclimation was started on different days for the different groups to ensure a temperature acclimation period of 14 days for each group of fish. For practical reasons temperature preference of the 18 groups of fish acclimated to three different temperatures could not be tested in random order. Instead pre-experimental treatments and temperature preference tests were first completed for the six groups of fish acclimated to 18°C and then for the six groups acclimated to 22°C and the six groups acclimated to 28°C.

#### Temperature preference tests

**Figure 2:**
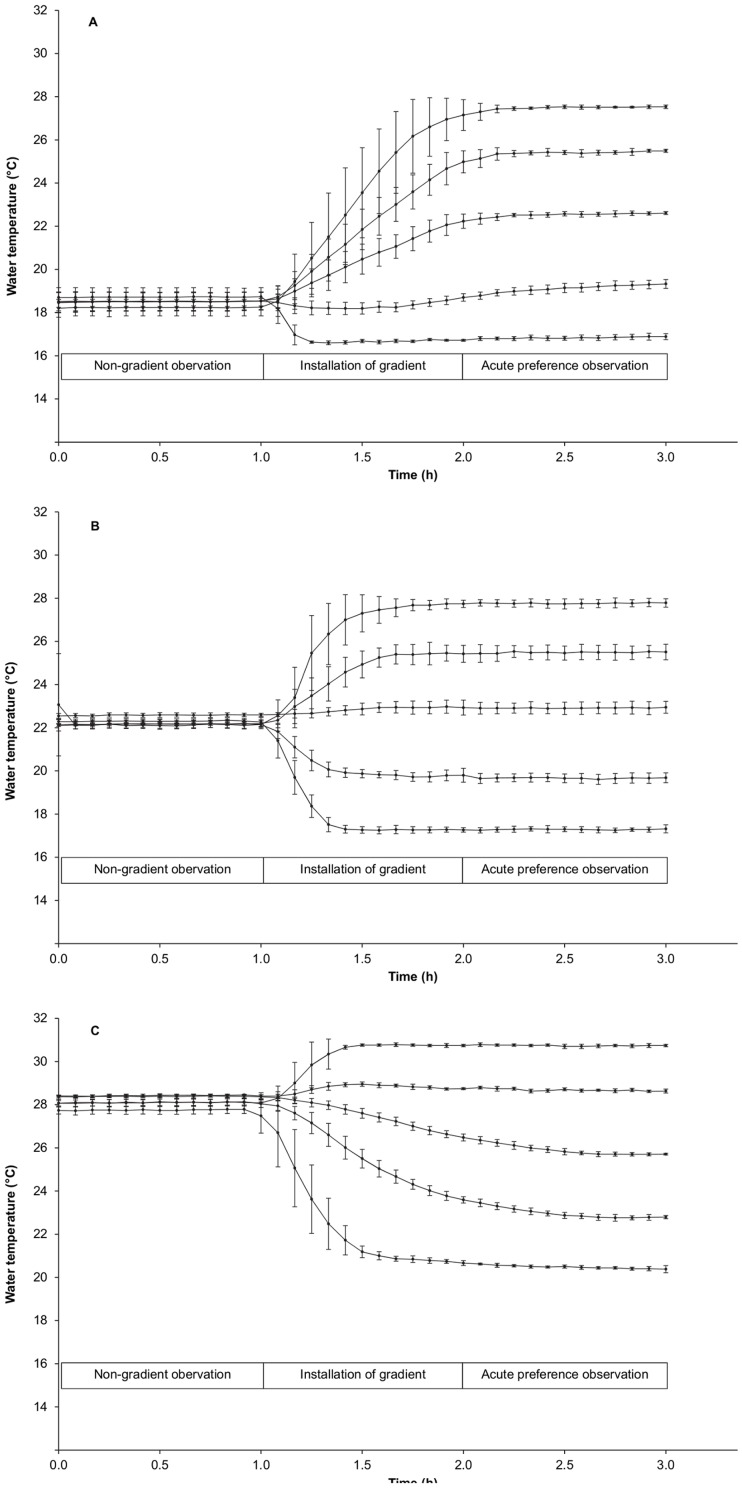
Water temperatures in the preference chamber during the preference tests for the three acclimation temperatures. Mean (SD, n = 6) water temperatures in the five temperature zones during the non-gradient observation, installation of the temperature gradients and acute preference observations are presented for the tests conducted with fish acclimated to 18°C (A), 22°C (B) and 28°C (C). Water temperatures during the 24 h preference observation equalled the water temperatures during the acute preference observations (not shown).

In total 18 temperature preference tests were performed; six tests for each of three different acclimation temperatures. Each test was conducted in a period of three days: On Day 1 at 16:00, coinciding with the 14^th^ day of the pre-experimental temperature acclimation period, a group of fish (n = 16) was transferred to the preference chamber. Fish were distributed over the *swimming channel* of the preference chamber by placing two fish in each of the eight compartments. Fish were then allowed to acclimate to the new conditions until 12:00 the next day (20 hours). On Day 2 from 12:00 to 13:00 the distribution of the fish over the preference chamber in absence of a temperature gradient was video recorded. This so-called ‘non-gradient observation’ served to detect any preference of fish for locations in the preference chamber caused by other factors than differences in water temperature (non-random use). Until then the water temperature in the entire preference chamber had been kept equal to the temperature to which the group of fish was acclimated (either 18, 22 or 28°C). After completion of the non-gradient observation the heating and cooling were switched on to install the temperature gradient in the preference chamber. Once the temperature gradient had been installed the distribution of the fish over the preference chamber was video recorded during one hour (14:00 to 15:00) to measure the acute temperature preference. The distribution of the fish was video recorded again 24 hours later (Day 3, 14:00 to15:00) to measure the 24 h temperature preference. The test was terminated at 15:00 on Day 3. The experimental procedure was identical for all tests. Fish acclimated to 18 and 22°C were exposed to a temperature range of 17–20–23–26–28°C and the range for the fish acclimated to 28°C was 20–23–26–28–31°C. During each test, water temperature was automatically measured and recorded every five minutes in the centre of each compartment at the inner wall of the s*wimming channel* of the preference chamber (Voltcraft K204). Temperature profiles during the tests are presented in Fig. 2A, B and C. Tests were performed under low (30 lux), constant light (24 hours per day) to enable video recording. Water was continuously recycled over the water storage tanks to ensure equal hydraulic conditions throughout the entire test period. Dissolved oxygen concentration (Hach Lange Multimeter), pH (Hach Lange Multimeter) and salinity (WTW Cond 315i) were measured at the end of each test in the *swimming channel* in the zone with the highest temperature. Mean (SD) dissolved oxygen concentration was 7.22 (0.17), pH ranged from 8.23 to 8.57 and mean (SD) salinity was 27.0 (0.9) g/L. In addition, dissolved oxygen concentrations were measured at 24 equally separated points in the *swimming channel* once for each of the two temperature ranges. In the 17–20–23–26–28°C range the dissolved oxygen concentration ranged from 7.2 to 8.3 mg/L, corresponding with an oxygen saturation range of 84 to 91%. In the 20–23–26–28–31°C range the dissolved oxygen concentration ranged from 6.6 to 7.7 mg/L, corresponding with an oxygen saturation range of 81 to 91%.

#### Data acquisition and statistics

For each test the distribution of fish over the preference chamber was observed by video recordings during three periods of one hour to determine non-gradient preference, acute temperature preference and 24 h temperature preference. The analysis of the video recordings was the same for each of the three recorded observations. For each recording the number of fish in each of the eight compartments was counted from screen shots at seven time points: 0, 10, 20, 30, 40, 50 and 60 minutes. In case a fish occupied two adjacent compartments, the fish was assigned to the compartment in which its head was positioned. For each compartment the total fish count was determined by summing the counts for each of the seven time points.

To obtain a measure of the relative usage of compartments, the total fish count for each compartment i 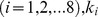, was divided by the grand total of counts 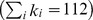, to obtain a set of 8 fractions 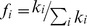 which together sum to 1 and are therefore referred to as a composition, indicated by the vector 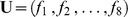.

An appropriate statistical framework for handling compositional data is to replace the observed proportions 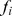 with a set of ratios by choosing the observed fraction of one particular component as the denominator by which all other fractions are divided. In this way, the unit sum constraint on the composition is broken [16]. By taking natural logarithms of these ratios, we obtain a vector 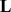 of log-ratios:
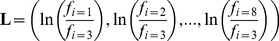
(1)The results of the analyses do not depend upon the choice of denominator, and a version of the central limit theorem exists stating why random variation in log-ratios can often be assumed to be normally distributed [16], [17]. For hypothesis testing and statistical modelling, multivariate regression models and MANOVA can be used (if the assumption of multivariate normality for the log-ratios is not too strongly violated) or randomisation tests [18], [19].

The usage of fish of compartments with different temperature zones *j* (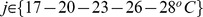 or 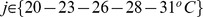) was measured by summing the usage fractions over the compartments with the same temperature: 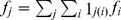, where 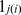 is an indicator function allocating a compartment to the corresponding temperature zone (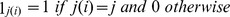). A set of log-ratios **P** is then obtained per test run, which describes the usage profile of fish over the temperature zones, by choosing one of the 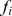
's as a denominator and taking logarithms (as in Eqn. 1). However, because the number of compartments differ per temperature zone, an actual measure of *preference* was obtained by dividing the usage fraction 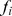 by the availability of temperature zone *j*, 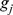. For the acclimation temperatures 18 and 22°C: 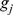 = 1/8 for temperature zones 17 and 28°C and 1/4 for temperature zones 20, 23 and 26°C. For the acclimation temperature 28°C: 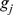 = 1/8 for temperature zones 20 and 31°C and 1/4 for temperature zones 23,26 and 28°C. This yields a new set log-ratios, denoted by the vector **Q**, where the vector of log-ratios of availability is subtracted from the log-ratio vector **P**. For example for test runs with acclimation temperatures 18 and 22°C:
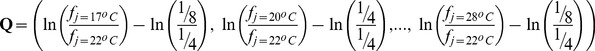
(2)The vectors **Q** (one for each test run) form the basic data for most of the statistical hypothesis testing in this paper. For each phase (acclimation, acute preference testing and 24 h preference testing) eighteen such vectors are available (6 test runs for each of the three acclimation temperatures), yielding a total of 54 vectors.

The following hypotheses are tested using the vectors **Q**:

1. H_o_: random use of the preference chamber, vs. H_1_: preference for certain temperature zones.
2. H_o_: no effect of acclimation temperature on preference for temperature zones, vs H_1_: an effect of acclimation temperature on preference for temperature zones.
3. H_o_: no effect of phase on preference for temperature zones, vs H_1_: an effect of phase on preference for temperature zones.

It is furthermore of interest to test whether there is evidence of a shift in preference from one phase to the next, for example from the acclimation phase to the acute preference phase. This can be done by conditioning, for each test run, upon the usage of fish in the acclimation phase. This is particularly of interest if there is evidence of non-random use of the preference chamber in the acclimation phase. Conditioning upon the usage fractions in the acclimation phase is done by element-wise subtraction of the log-ratios in the successive stages, for example:
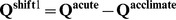
(3a)

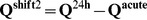
(3b)

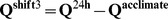
(3c)We note that fish acclimated to 18 and 22°C were exposed to a slightly different temperature gradient in the preference chamber than the fish acclimated to 28°C: 17, 20, 23, 26, 28°C versus 20, 23, 26, 28, 31°C. Treatments were therefore compared for the shared exposure temperatures: 20, 23, 26 and 28°C.

Once overall evidence of non-random use and treatment effects has been assessed using the MANOVA procedures, the next step is to rank habitats from least to most preferred. This can be done by computing a cross-table with pairwise differences between matching log-ratios of usage and availability of temperature zones, and counting the number of times a particular temperature zone has been observed to be preferred over other temperature zones (e.g. see table 1 in Aebischer et al 1993 [18]). The ranking of preference for temperature zones was done separately for the three treatments. The statistical significance of the pairwise differences in mean relative preference between temperature zones was assessed using Wilcoxon rank sum tests (with n = 6 replicate test runs per treatment).

All statistical analyses were performed in R. The R-code to analyse data from the preference chamber and a data file are provided as supporting information files (Table S1 and S2).

### Growth experiment

#### Experimental design

**Figure 3:**
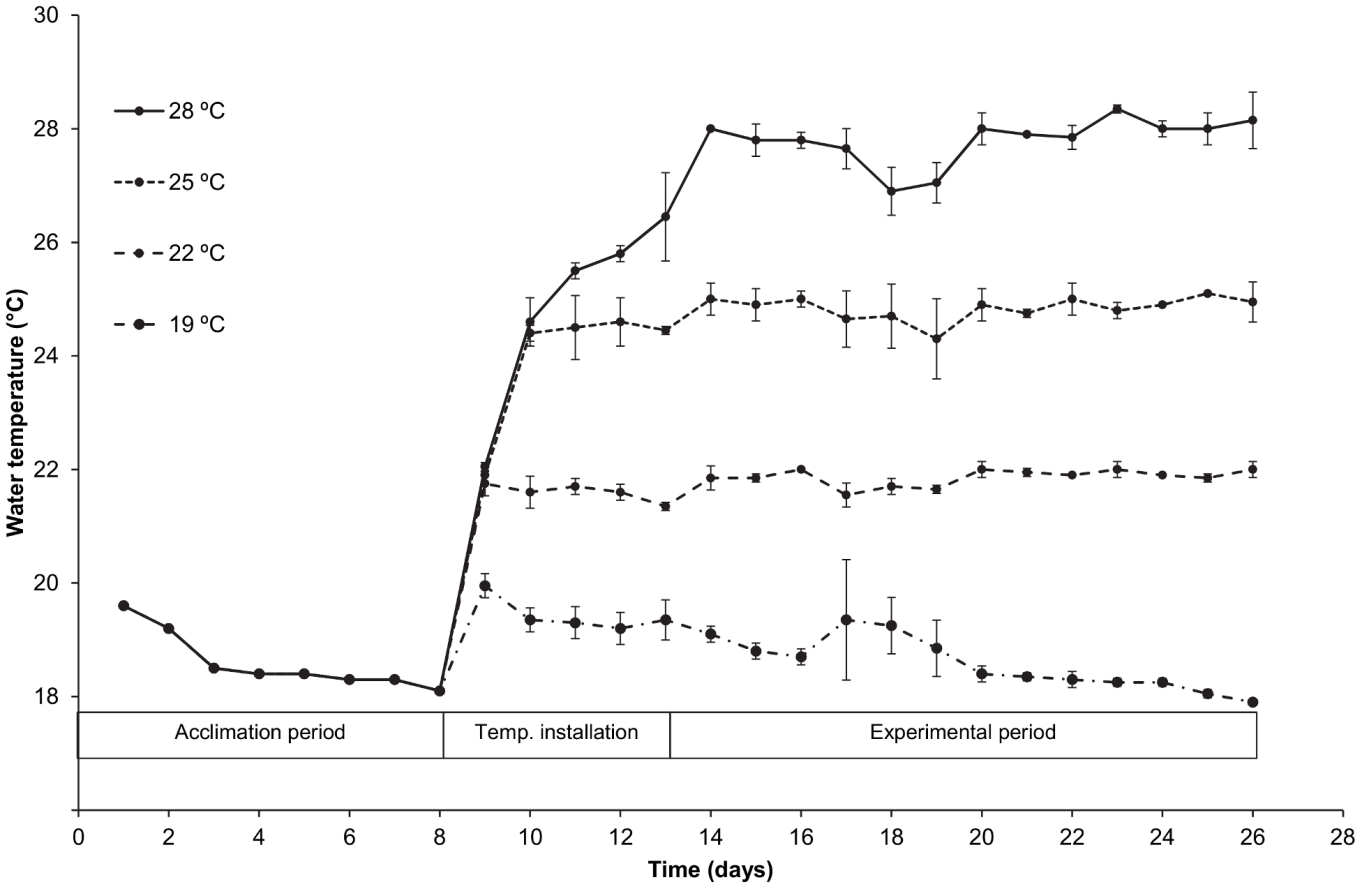
Water temperatures during the growth experiment. For each temperature treatment the mean (SD, n = 2) water temperature during the acclimation, installation of temperatures and the experiment is presented. Overall mean temperature per treatment during the experiment (n = 26) differed significantly among all treatments (ANOVA, p<0.0001).

On day 1, Dover sole (N = 128) with a mean (SD) weight of 33.3 (7.1) g, corresponding to a total length range of 12.8 to 17.2 cm, were randomly distributed over eight aquaria (70×70 cm, 50 L, 16 fish per aquarium). Aquaria were part of the recirculating system described above, with an initial water temperature of 18.6±0.5°C. Treatments consisted of four different water temperatures (19, 22, 25 and 28°C, Fig. 3) and were randomly assigned to the eight aquaria (two aquaria for each treatment). Six out of eight aquaria were equipped with a thermostatically controlled heater to heat the water to the desired temperature. After stocking fish were allowed to acclimate to the new conditions until day 8. Starting on day 9, treatments were installed by gradually increasing water temperatures (∼2°C per day) until the desired water temperatures were reached in all aquaria (day 14 for the 28°C treatment, Fig. 3). The desired temperatures were then maintained for 12 days until termination of the experiment on day 26. Water temperature was measured daily in each aquarium (Hanna HI93510). Dissolved oxygen concentration was measured daily in each aquarium (Hach Lange HQ40 multimeter) from day 9 to 26. Total ammonia (NH_3_+NH_4_^+^) concentrations, measured in all aquaria on day 15 and 22 (photometrically, Hach Lange DR2800) were below 1.0 mg N/L.

#### Feeding and measurement of feed intake

Fish were fed small live rag worms (*Nereis diversicolor*, Topsy Baits, The Netherlands). Every morning at 9.00 am, starting on day 1, each aquarium was supplied with a pre-determined amount of rag worms (50–105 g wet weight, based on feed intake on the previous day). Before supplying rag worms, the uneaten rag worms supplied on the previous day were removed from each aquarium and weighed. Fish were fed slightly in excess to ensure maximum feed intake and to avoid variation in feed intake among individual fish within tanks caused by domination of the feed supply by a limited number of individual fish. Feed load was determined daily for each individual aquarium based on the feed intake on the previous day. Day 1 to 7 were used to reach maximum feed intake in all aquaria. The daily feed intake per aquarium was determined by subtracting the recovered amount of rag worms from the amount supplied on the previous day. Rag worms could not escape from the aquaria. Rag worm biomass was determined after removal of excess water in a standardized manner.

#### Fish health

To investigate the effect of water temperature on fish health, three fish were sampled at day 26 from both the 19°C and 28°C temperature groups for bacteriological diagnostics by the Fish Diseases Laboratory of the Central Veterinary Institute of Wageningen UR, Lelystad, The Netherlands. After euthanizing the sole by an overdose of 2-phenoxy ethanol and subsequent decapitation, samples were taken from the skin, head and fin with sterile disposable plastic loops. These were inoculated onto Brain Heart Infusion (BHI) agar (Oxoid) with 5% (v/v) sheep blood, and onto Marine agar (Oxoid). From each fish, the liver, spleen, and kidney were externally disinfected with a heated scalpel. In each organ a sterile incision was made and samples were taken from inside the incision with sterile disposable plastic loops. Samples were inoculated onto BHI agar with 5% (v/v) sheep blood. The inoculated agar plates were incubated at 22°C for maximum 7 days, and were examined for bacterial growth at days 1, 2, 5 and 7. If there was no bacterial growth after 7 days, or, if there was a multi-bacterial growth the agar plates were no longer incubated. If there was a predominant or pure culture of bacteria from organs and/or skin lesions, the bacteria were typed according to Austin and Austin (1989) [20] and Barrow and Feltham (1993) [21] and by 16S RNA typing. A 16S rDNA sequence analysis was performed adapted from Schuurman et al (2004) [22]. Files were analysed by MicroSeq 500® Bacterial Identification Kit or Sequence analyser using either the Microseq® program database or the NCBI Blast database to compare the obtained sequences with sequences of different bacteria species. Colonies were counted after 4 days, for a semi-quantitative indication on bacteria in the sampled fish.

#### Data acquisition and statistics

Fish were weighed individually to the nearest 1 g on day 1 and 26 (Mettler PM 34 Delta range). Total fish biomass was measured on day 14 in each aquarium.

Total feed intake, specific growth rate and feed conversion ratio were calculated for the period in which the treatments were fully installed (day 14 to 26).

The daily feed intake per aquarium was divided by the number of fish in the aquarium to account for differences in numbers of fish among aquaria, resulting in a daily feed intake per fish for each aquarium. For each aquarium the total feed intake per fish (TFI) was determined by summation of daily feed intake per fish. Total feed intake per fish and biomass increase per aquarium were used to calculate feed conversion ratio (FCR) as follows:
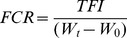
Where FCR = feed conversion ratio (g/g), TFI = total feed intake (g/fish), W_t_ = mean individual weight at day 26 (g) and W_1_ = mean individual weight at day 14 (g).

Specific growth rate (SGR) was calculated as follows:
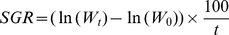
where SGR = specific growth rate (%/d), W_t_ = mean weight at day 26 (g), W_0_ = mean weight at day 14 (g) and t = number of days.

Aquaria were inspected for mortalities twice daily. Dead fish were removed upon detection.

Initial and final individual weights per tank were calculated from the individual weight measurements (n = 16/tank). Initial and final individual weights were then expressed as mean per treatment (n = 2). Total feed intake per fish (TFI), specific growth rate (SGR) and feed conversion ratio (FCR) were expressed as mean per treatment (n = 2). Mean values per treatment were tested for significant differences among the treatments by one-way ANOVA in SAS 9.2. Only in case significant treatment effects were detected, a least significance difference (LSD) post-hoc analysis was used to estimate the level of significance between mean values. For both ANOVA and LSD analysis the fiducial limit was set at 5%.

The relations between temperature and SGR, TFI and FCR were analyzed by second order polynomial regression analysis in SAS 9.2.

### Predicted relations between thermal acclimation and thermal preference

Preferred temperature has been reported to either increase with acclimation temperature until stabilizing at the *final preferedum* [2] (hereafter referred to as category 1) or always to correspond to the *final preferendum* irrespective of acclimation temperature [5] (hereafter referred to as category 2). For Dover sole we predicted the relation between acclimation temperature and preferred temperature in accordance with both categories. For category 1 the infliction point or the *final preferendum* was set at the optimal temperature for growth measured in the growth experiment. The slope of the line before the infliction point is unknown. For category 2 the preferred temperature was fixed at the measured optimal growth temperature, independent from the acclimation temperature.

## Results

### Thermoregulatory behaviour

**Figure 4:**
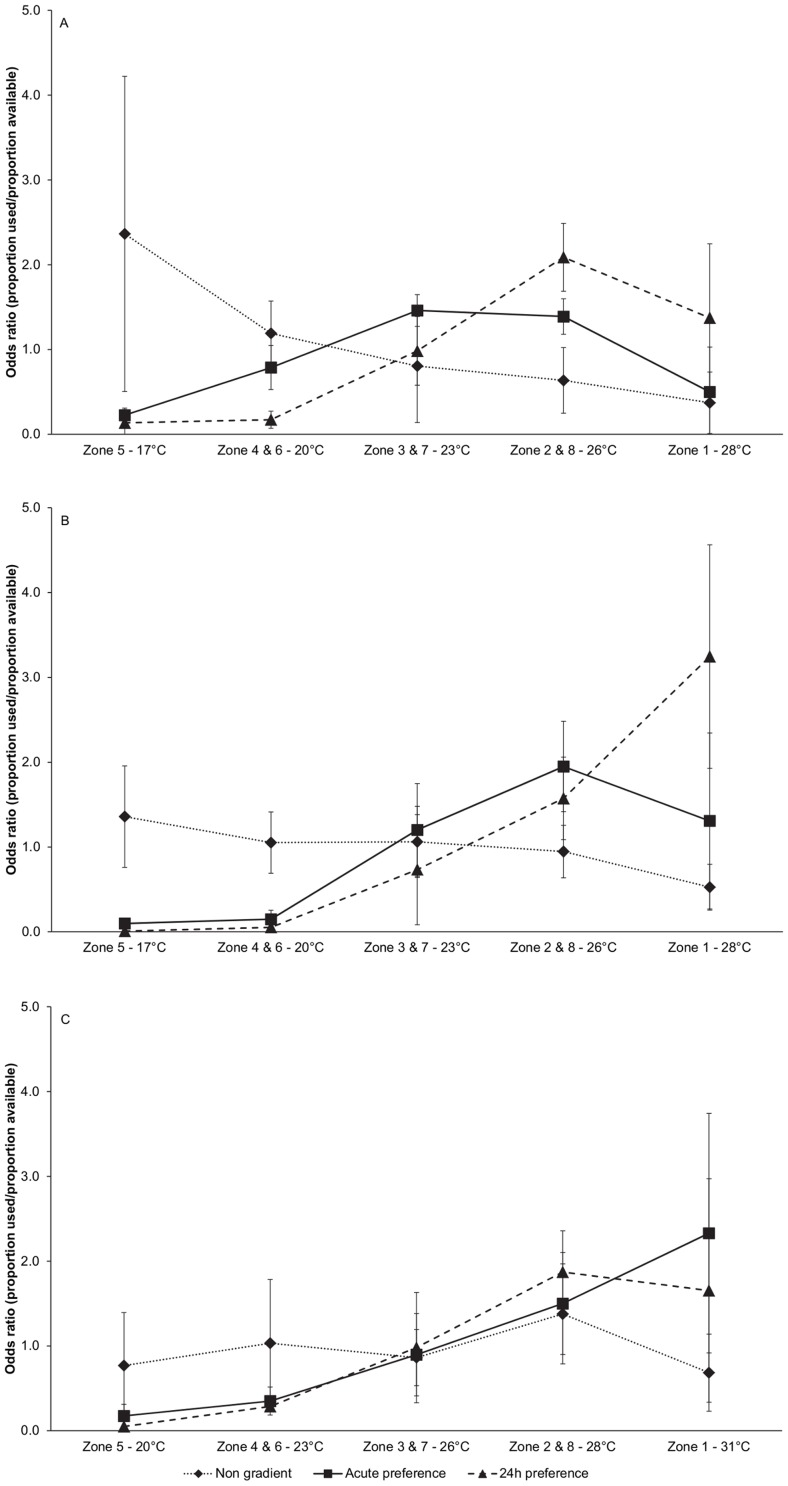
Fish distribution over the preference chamber. Fish distributions during non-gradient, acute preference and 24 h preference observations are expressed as odds ratios (proportion of temperature zones used dived by the proportion of temperature zones available) and are presented for fish acclimated to 18°C (A), 22°C (B) and 28°C (C). Fish distribution was non-random during all non-gradient observations (MANOVA, p = 0.002) without differences among temperature acclimation treatments (MANOVA, p = 0.42). Fish distribution observed during acute preference observations differed significantly from the non-gradient observations (MANOVA, p<0.001). Fish distribution during 24 h preference observations differed significantly from the non-gradient (MANOVA, p<0.0001) as well as the acute preference observations (MANOVA, p<0.002). Among acclimation temperature treatments fish distribution differed during acute preference observations (MANOVA, p = 0.01) but not during the 24 h preference observations (MANOVA, p = 0.21).

During the non-gradient observations fish distribution across the preference chamber was found to be non-random (MANOVA, p = 0.002) for all three treatments (Figs. 4A, 4B, 4C), indicating that in the absence of temperature differences fish show some preference for given areas of the preference chamber. Among the three treatments no differences in fish distribution during the non-gradient observations were detected (MANOVA, p = 0.42).

Installing a temperature gradient in the chamber lead to an immediate (acute) significant change in distribution of fish in all treatments compared to the non-gradient observations (MANOVA, p<0.001) (Figs. 4A, 4B, 4C). In addition, in the acute preference tests, the distribution of fish across the temperature zones in the chamber were significantly different among the different acclimation treatments (MANOVA, p = 0.01). Fish acclimatised at lower temperatures showed a peak occurrence at lower temperatures compared to fish acclimatised at higher temperatures (Figs. 4A, 4B, 4C).

The distribution of fish observed 24 hours after installation of the temperature gradient differed from the distribution in the absence of a temperature gradient (non-gradient, MANOVA, p<0.0001) as well as from distribution during the acute tests (acute, MANOVA, p<0.002) (Figs. 4A, 4B, 4C). In contrast to the acute preference test, the distributions of fish across the temperature ranges in the chamber were not different among the different acclimation treatments (MANOVA, p = 0.21).

### Acute and 24 h thermal preference

Acute and 24 h temperature preferences were assessed by ranking of use of the different temperature zones in the preference chamber (Table 2). The acute preference of fish acclimated to 18°C was 23°C, although the use of the 23°C zones was not significantly different from the 26 and 28°C zones. The use of the 17 and 20°C zones was significantly lower than the 23 and 26°C zones but not different from the 28°C zone. After 24 h the preferred temperature of the fish acclimated to 18°C had increased from 23°C to 26°C, although the use of the 26°C zones was not significantly different from the 23 and 28°C zones.

**Table 2:** Ranking of the use of the temperature zones in the preference chamber (PC) by Dover sole acclimated to 18, 22 and 28°C, directly after installation of the temperature gradient (acute preference) and 24 h thereafter (24 h preference).

	Acute preference			24 h preference		
Acclimation temperature (°C)	18	22	28	18	22	28
Temperature in PC (°C)						
30.7 (0.1)	-	-	4^b^	-	-	3^b^
27.9 (0.5)	1^abc^	2^ab^	3^b^	3^b^	4^c^	4^b^
25.6 (0.3)	3^c^	4^b^	2^b^	4^b^	3^b^	2^b^
22.8 (0.3)	4^c^	3^b^	1^a^	2^b^	2^bc^	1^b^
19.7 (0.6)	2^b^	1^a^	0^a^	1^a^	1^a^	0^a^
17.1 (0.3)	0^a^	0^a^	-	0^a^	0^a^	-

Ranks with different superscripts are significantly different (t-test, p<0.05). 4 = most used, 0 = least used.

The acute preference of fish acclimated to 22°C was 26°C, higher than the acute preference of fish acclimated to 18°C. The use of the 26°C zones was however not significantly different from the 23 and 28°C zones. After 24 h the preferred temperature of fish acclimated to 22°C had increased to 28°C. The use of the 28°C zone by these fish was significantly higher than the use of any of the other zones.

The acute preference of fish acclimated to 28°C was 31°C, again higher than the acute preferences of the other two groups. The use of the 31°C zone was however not significantly different from the 28 and 26°C zones. After 24 h the preferred temperature of the fish acclimated to 28°C had decreased to 28°C, although the use of the 28°C zones was not significantly from the 23, 26 and 31°C zones.

The ranking of temperature zone shows that acute preference increases with acclimation temperature. It also shows that after 24 h there is little effect of the pre-experimental temperature acclimation on temperature preference. Both results agree with the already presented evidence that acclimation affects the acute distribution of fish over the preference chamber but not the distribution 24 h after installation of the temperature gradient.

### Thermal optimum for growth

**Figure 5:**
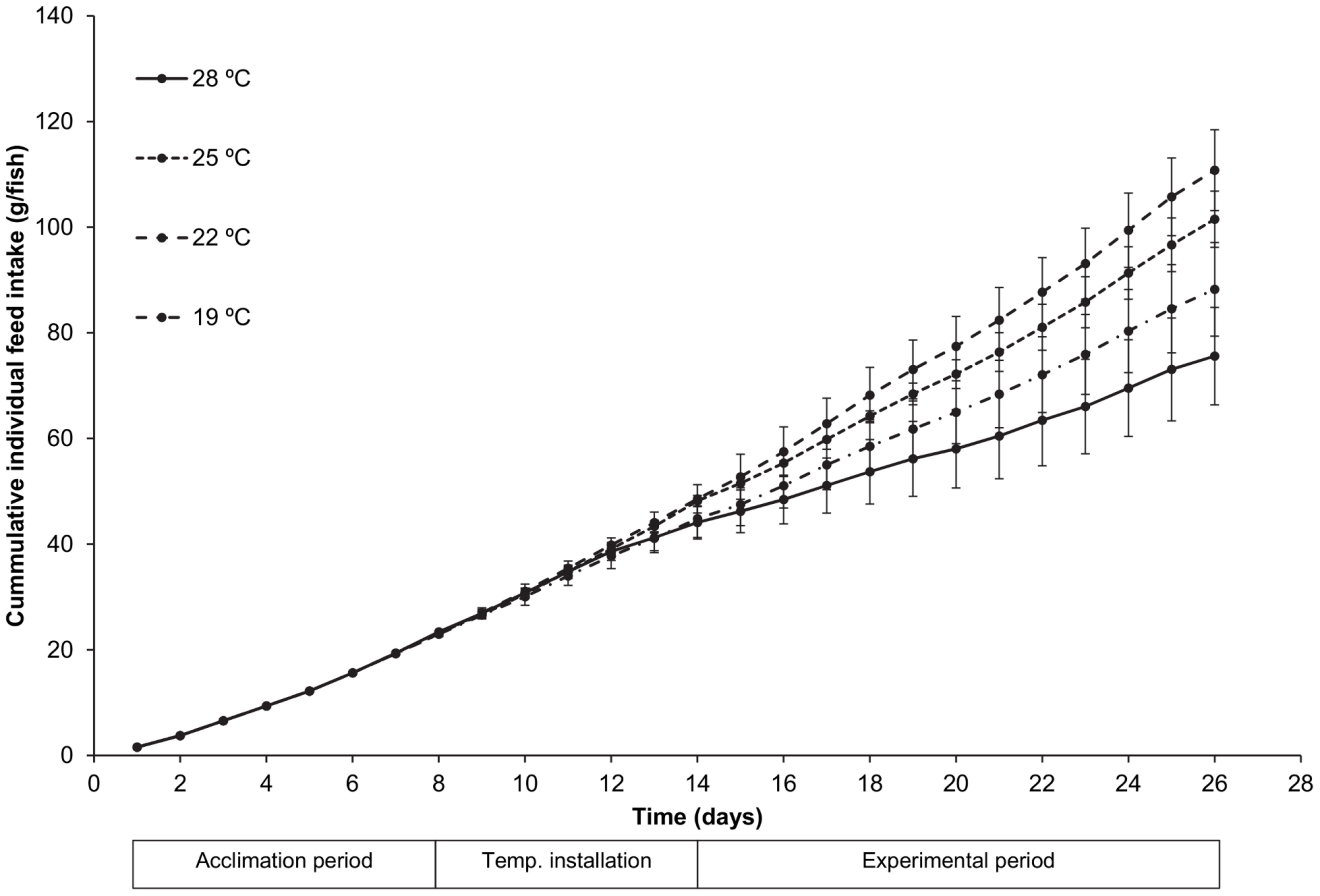
Cumulative food intake of juvenile Dover sole (*S.* *solea*) in relation to water temperature. Mean (SD, n = 2) cumulative food intake during acclimation, installation of temperatures and the experimental period are presented per water temperature treatment.

**Figure 6:**
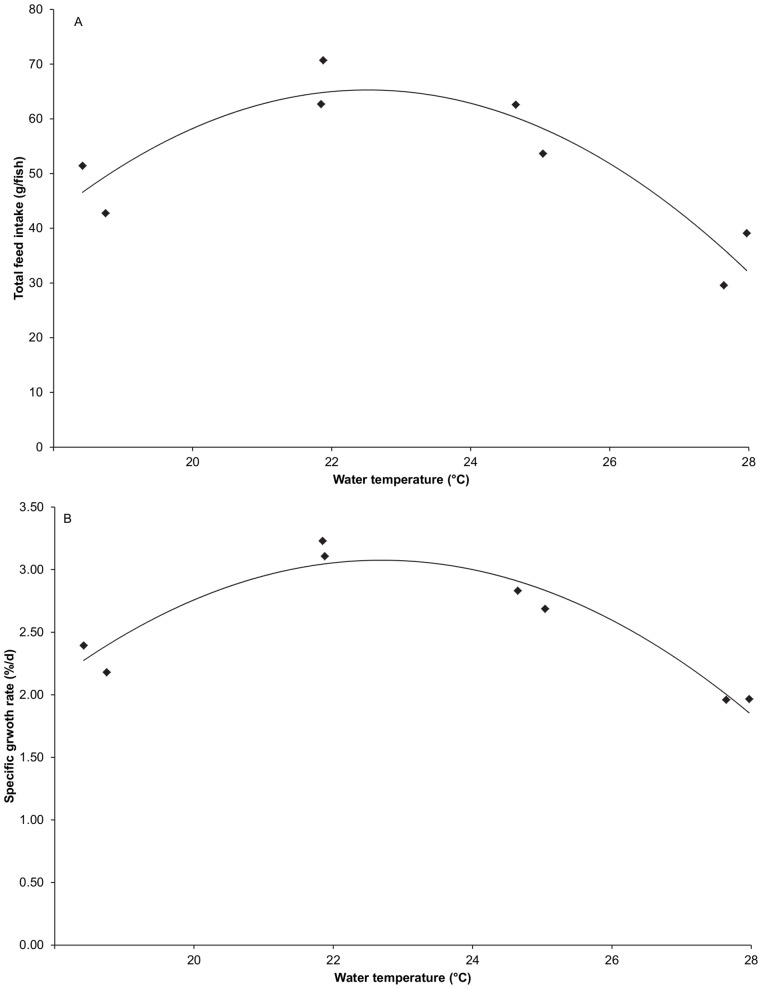
Total feed intake and specific growth rate of juvenile Dover sole (*S.* *solea*) in relation to water temperature. Total feed intake (A) (TFI, g/fish) peaks at 22.5°C. Total feed intake (g/fish) TFI = −1.111.T^2^+50.T−498 (polynomial regression analysis, model p-value = 0.012, r^2^ = 0.77). Specific growth rate (B) (SGR, %/d) peaks at 22.5°C. SGR = −0.044.T^2^+1.99.T−19.5 (polynomial regression analysis, model p-value = 0.002, r^2^ = 0.89). T = water temperature (°C).

Mean cumulative feed intake per fish is shown in Fig. 5 for the four temperature treatments. Differences among treatments started to develop during installation of the temperature treatments and became more and more pronounced over time. This ultimately results in significant differences in total feed intake among treatments. Significant differences among treatments were also observed for specific growth rate and feed conversion ratio (Table 3). The highest total feed intake and specific growth rate and the lowest feed conversion ratio were all observed in the 22°C treatment. Specific growth rate, total feed intake and feed conversion ratio were all found to be significantly related to water temperature (Fig. 6A and 6B; Table 4). The regression analysis yielded very similar optimal temperature estimates for total feed intake (22.5°C), specific growth rate and feed conversion ratio (both 22.7°C).

**Table 3:** Results of the growth experiment.

Treatment	Temperature	[O_2_]	Weight Day 1	Weight Day 13	Weight Day 26	TFI	SGR	FCR	Survival
(°C)	(°C)	(mg/L)	(g)	(g)	(g)	(g/fish)	(%BW/d)		(%)
19	18.6 (0.5)^a^	7.2 (0.0)^a^	33.9 (0.27)	42.3 (0.27)	49.3 (1.28)	47.1 (6.1)^ac^	2.29 (0.15)^a^	6.7 (0.1)^ac^	100 (0)
22	21.9 (0.2)^b^	6.8 (0.1)^b^	33.0 (3.84)	43.0 (3.83)	56.3 (4.38)	66.7 (5.7)^b^	3.17 (0.09)^b^	5.0 (0.2)^b^	93 (9)
25	24.8 (0.3)^c^	6.2 (0.3)^c^	33.3 (0.82)	41.9 (1.41)	52.0 (1.07)	58.1 (6.3)^ab^	2.76 (0.10)^c^	5.8 (0.4)^ab^	97 (4)
28	27.8 (0.4)^d^	6.3 (0.1)^c^	32.8 (2.86)	39.8 (4.02)	44.6 (4.52)	34.4 (6.7)^c^	1.96 (0.00)^d^	7.2 (0.7)^c^	94 (0)
p-value	<0.0001	<0.0001	0.97	0.73	0.09	0.02	0.001	0.02	0.59
LSD1)	0.21	0.17	-	-	-	17.3	0.28	1.14	-

Mean (SD) values for water temperature (n = 26), dissolved oxygen concentration ( [O_2_], n = 26 ), individual weights at days 1, 14 and 26 (n = 2), total feed intake (TFI, n = 2), specific growth rate (SGR, n = 2) and feed conversion ratio (FCR, n = 2).

1) LSD = least significant difference.

Mean values with different superscripts are significantly different (ANOVA, p<0.05).

**Table 4:** Results of the second order polynomial regression for specific growth rate (SGR), total feed intake (TFI) and feed conversion ratio (FCR).

Dependent variable	Quadratic component	p-value	Linear component	p-value	Constant	p-value	R-square
SGR	−0.044	<0.001	1.99	<0.001	−19.5	0.002	0.89
TFI	−1.111	0.006	50.0	0.007	−498	0.012	0.77
FCR	0.0828	0.003	−3.77	0.003	48.0	0.002	0.82

### Prediction relations between thermal acclimation and thermal preference

**Figure 7:**
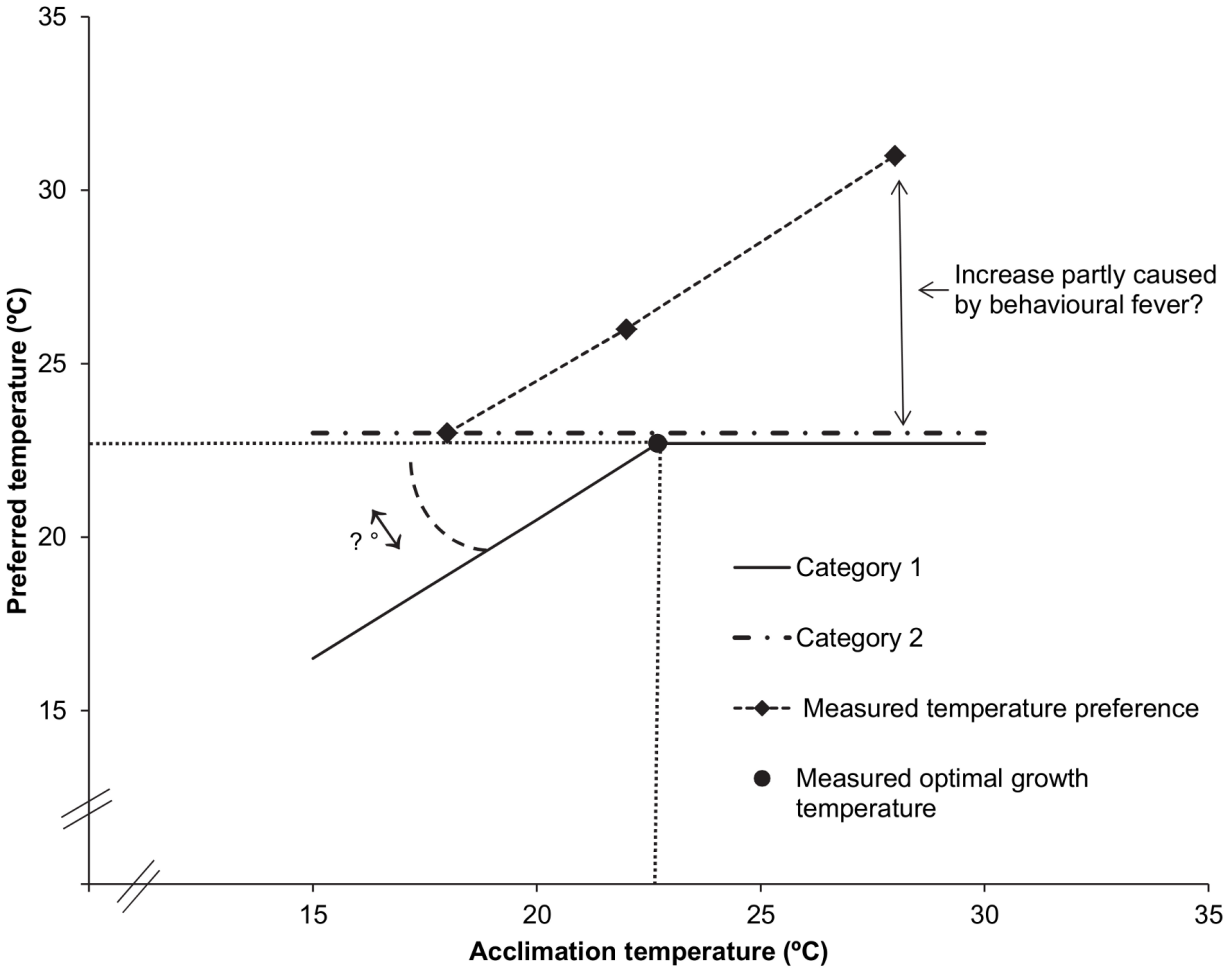
Predicted and measured relations between acclimation temperature and preferred temperature for juvenile Dover sole (*S.* *solea*). For fish in category 1 the preferred temperature increases with acclimation temperature until they equal each other at the *final preferendum*. Beyond the *final preferendum*, here set at the measured optimal growth temperature, the preferred temperature stabilises. For fish in category 2 the preferred temperature is independent from the acclimation temperature and lies close to the *final preferendum* and optimal growth temperature. The measured acute preference increases with acclimation temperature.

The relations between acclimation and preferred temperatures as predicted for category 1 and category 2 fish and as measured in the preference tests are presented in Fig. 7.

### Fish health

No bacterial growth was observed from the internal organs of fish from the 19°C treatment. The samples from the skin, head and fin of this group resulted in a multi-bacterial growth of on average five colonies per sample. *Pseudoalteromonas* species was identified.

Light bacterial growth with on average five colonies per three platings was observed from the internal organs of fish from the 28°C group. The samples from the skin, head and fins resulted in multi-bacterial growth with on average 67 colonies per sample. *Vibrio ichthyoenteri/scophthalmi* were found both in the external and internal samples. *Pseudoalteromonas* species were found only in the external samples (skin, head and fins).

Mortalities occurred in 4 out of 8 aquaria, but none in the aquaria kept at 19°C. However, no significant treatment effect on survival was detected (Table 3).

## Discussion

### Experimental set up

We observed non-random use of the preference chamber in absence of a temperature gradient. Despite our efforts to create a homogenous environment, the distribution of the fish over the preference chamber was apparently affected by factors we were unaware of and unable to control. We effectively accounted for this non-random use by considering the change in the distribution of fish following the installation of the temperature gradient rather than the actual distribution of fish in the temperature gradient. We conclude that in animal preference experiments, the distribution of the animals in the preference test facility should also be measured in absence of the exposure gradient to detect the presence of non-random use.

As water temperature affects oxygen solubility, the installation of a temperature gradient may also result in the presence of an oxygen gradient, where the highest temperatures correspond with the lowest dissolved oxygen concentrations. As thermal tolerance is correlated to availability of oxygen [23], an oxygen gradient present within a thermal gradient may interact with measured temperature preferences. Dissolved oxygen concentration measurements throughout the entire preference chamber indeed showed the presence of an oxygen gradient. The differences in dissolved oxygen concentration and especially saturation (81–91%) are small and all levels are within physiological ranges. Nevertheless, an effect of oxygen on preference for locations cannot be entirely excluded. If fish prefer locations with higher dissolved oxygen concentrations over locations with lower dissolved oxygen concentrations, we possibly underestimated the preference for higher temperatures within the offered temperature gradients.

Senegal sole (*Solea senegalensis*) display a clear daily activity rhythm, with the highest activity during night hours [24]. Daily activity rhythms have not been experimentally assessed in Dover sole but these could be similar to Senegal sole as for the nocturnal nature of both species [25], [26]. If so, our measurements of acute and 24 h preference took place during periods of relatively low activity of the fish. Whether this significantly influenced our results is unclear and demands further research. As diurnal activity and endocrine (cortisol) rhythms in Senegal sole persisted under constant light conditions [24], we do not expect that results of our preference test were influenced by conducting them under constant light.

### Behavioural fever and fish health

Ectothermic vertebrate species injected with bacteria have been shown to prefer higher temperatures, a phenomenon referred to as behavioural fever that is, like physiological fever, believed to combat infections [27]. Behavioural fever has not been shown in Dover sole nor, to the best of our knowledge, any other related marine fish species. However, if the experimental fish suffered from an infection, the large difference between the measured optimal growth temperature of 22.7°C and the acute preference for 31°C measured in fish acclimated to 28°C could (partly) have resulted from behavioural fever. The health tests of fish in our growth experiment suggest that bacterial growth from the inside and outside of the sole was more pronounced in fish reared at 28°C than reared at 19°C. The bacteria observed are known as a commensal gut bacterium of flatfish (*Vibrio ichthyoenteri/scophthalmi*, [28]) and a genus found in the marine environment (*Pseudoalteromonas* [29]) and show higher growth rate at 28°C than at 19°C [30], [31]. These bacteria are not considered as primary pathogenic to fish although their health impact when occurring in higher quantities is unknown. More standardized quantitative bacteriology and longer term rearing of Dover sole at different temperatures would be required to fully investigate the effect of water temperature on health.

A confounding effect of behavioural fever on the observed thermal preferences cannot be entirely excluded. However, it may only partly explain the large difference between the measured optimal growth temperature of 22.7°C and the acute preference for 31°C measured in Dover sole acclimated to 28°C as the maximum increase in preferred temperatures observed in experimentally infected teleost fish, amphibians and reptiles is 5°C [32].

### Thermoregulatory behaviour

We predicted a behavioural response of Dover sole when exposed to a temperature gradient. Their distribution in the preference chamber was indeed significantly affected by the installation of the temperature gradient. As the fish used in this experiment were progenies of wild-caught breeders from the North Sea, their thermal tolerance was most likely equal to the natural thermal niche. Under natural conditions the thermal habitat ranges from approximately 5 to 27°C, with marked differences among life stages [33]. Field studies in the North Sea also showed that Dover sole respond to changes in temperature. In severe winters, they leave their normal wintering areas when the temperature drops below 5°C and move to warmer water in the western North Sea [34], [35], but there are no indications that juvenile sole avoid the coastal waters when temperatures reach their seasonal maximum [36], [37].

Our preference tests results and previous field observations jointly taken, lead to the conclusion that Dover sole is a thermo-sensitive fish species capable of detecting temperature differences and behavioural thermoregulation.

### Acute thermal preference

Two different relations between acute temperature preference and thermal history have been reported. First, acute temperature preference of fish has been reported to be strongly influenced by previous thermal acclimation. Acute preferences then increase with acclimation temperature before stabilising at the *final preferendum* [2]. The acute temperature preference of fish has also been reported to be more or less equal to the *final preferendum*, irrespective of previous thermal acclimation [5].

In agreement with Reynolds and Casterlin (1979) [2], we indeed observed an increasing acute preference with increasing acclimation temperature, but in our experiment the preferred temperature never stabilised. Instead, the acute preference was always above the acclimation temperature, even for fish acclimated to 28°C (Fig. 7), suggesting that at 28°C the *final preferendum* had not yet been reached. The increase of the acute preference of fish acclimated to 22°C and 28°C as compared to fish acclimated to 18°C can probably not be entirely explained by behavioural fever [32] as discussed above. It thus appears that our observations on the acute thermal preference of Dover sole in relation to its thermal history are not entirely in accordance with current views on this relation [2],[5] (Fig. 7).

### 24 h-thermal preference

During prolonged, 24 h or more, exposure to a temperature gradient, fish have been reported to ‘gravitate’ to species specific *final preferenda* that are not influenced by previous thermal acclimation. The influence of previous acclimation is thought to diminish when fish re-acclimate in the temperature gradient [2]. We therefore predicted the 24 h preference of Dover sole to correspond to its *final preferendum* and to be unaffected by the different temperature acclimation treatments prior to the preference tests. We indeed observed that, in contrast to the acute preference observations, 24 h after their introduction, the distribution of the fish over the preference chamber was not significantly influenced by the pre-experimental temperature acclimation.

It has also been reported that long-term preferred temperatures are generally slightly higher than acutely preferred temperatures as long as the acclimation temperature does not exceed the *final preferendum* [9]. Our observations in sole acclimated to 18 and 22°C were in agreement with this. However, when acclimated to 28°C, the acute preference was higher than the 24 h- preference, suggesting that at 28°C the *final preferendum* had been exceeded. These observations combined indicate that based on the 24 h preference test the *final preferendum* of juvenile Dover sole lies between 22° and 28°C, which partly overlaps the predicted range of 20° to 25°C. In contrast to our prediction, the acute and 24 h preference tests did not result in the same estimate for the *final preferendum*.

### Thermal optimum for growth and thermal preference

Thermal preferences of fish have been found to coincide with thermal optima of physiological processes [3] including growth [38]. Based on this we predicted that the optimal growth temperature resulting from the growth experiment would equal the *final preferendum* resulting from both the acute and 24 h preference tests.

According to our growth experiment the optimal growth temperature of juvenile Dover sole (30–50 g) is 22.7°C. This result corresponds very well to, and provides further refinement of the previously reported optimal temperature range of 20 to 25°C [12].

No *final preferendum* was reached in the acute preference tests, suggesting that both the *final preferendum* and the optimal growth temperature exceed the highest experimental acclimation temperature of 28°C. This is however unlikely considering the significant reduction of feed intake and growth of fish reared at 28°C compared to fish reared at 22°C in the growth experiment. In addition, the lethal temperature of sole larvae has been reported to be 28°C [13]. Based on this we conclude that the optimal temperature for growth certainly does not exceed 28°C as suggested by the acute temperature preference tests.

The *final preferendum* yielded by the 24 h-preference tests, lying in the range between 22°and 28 °C, also did not provide an accurate estimate of the optimal growth temperature of 22.7°C according to the growth experiment. Clearly the use of temperature preference tests to determine optimal growth temperatures as suggested by Jobling (1981) [9] proved to be inapplicable to Dover sole. Considering that growth is the result of many different physiological processes, it appears that thermal preference is not directly related to the thermal optima for physiological processes in Dover sole. It has been suggested that in such cases evolutionary coadaptation of thermal preference and thermal optima is constrained and that thermal preferenda may reflect a primitive condition rather than an adaptation to the current thermal environment [4].

### Conclusions

We explored several aspect of the thermal biology of an ectothermic vertebrate. Our observations revealed that thermal preference and thermal optima for physiological processes may be unrelated in Dover sole, making it an interesting model species to further explore the relation between thermoregulatory behaviour and thermal physiology in ectothermic vertebrates.

We conclude that juvenile Dover sole are thermo-sensitive; they are capable of detecting temperature differences and behavioural thermoregulation. The optimal growth temperature is 22.7°C for juvenile Dover sole between 30 to 50 g, provided food availability is not limited. Acute temperature preference is affected by acclimation temperature. In contrast, 24 h temperature preference is not affected by acclimation temperature. Acutely preferred temperatures exceed the optimal growth temperature. It is clear that temperature preference cannot be used to estimate the optimal growth temperature of Dover sole.

## Supporting Information

Table S1 **Example R-code to analyse data from the preference chamber experiments.**
(DOCX)

Table S2 **Data file preference chamber.**
(CSV)
