# Supplementary material for: Thermal Preference of Juvenile Dover Sole (Solea solea) in Relation to Thermal Acclimation and Optimal Growth Temperature
Source: PLoS One. 2013 Apr 22;8(4):e61357. doi: 10.1371/journal.pone.0061357 (PMC3632572; doi:10.1371/journal.pone.0061357)
Supplement: Table S1 — Example R-code to analyse data from the preference chamber experiments. (DOCX) [file pone.0061357.s001.docx]

**Table S1. Example R-code to analyse data from the preference chamber experiments.**

Here, we provide example R-code for the statistical analyses of data from the preference experiments, with acclimation temperatures of 18 and 22 ^0^C. Fish acclimated to 18 and 22°C were exposed to a temperature range of 17-20-23-26-28°C. The example R-code

***# Step 1: read in the raw data: counts of numbers of fish per zone in the preference***

***# chamber, with column*** ***headings:***

*# Experiment: Indicator for experiment: 1,2,…,17,18*

*# Acclimatisatie.temp: Acclimation temperature of an experiment: the temperature of all*

*# Zones within the preference chamber during the acclimation phase*

*# Compartiment: Indicator for compartment within the preference chamber: 1,2,…8*

*# Temperatuur: Temperature of a compartment in the acute and 24 hour phases*

*# Fase: Phase of the experiment indicated by roman numerals I acclimation),II # (acute) or III (24 hours)*

*# T0, T10,…,T60: Count of numbers of fish at the start of the phase (T0) at 10 minutes*

*# from the start of the phase (T10),….,at 60 minutes from the start of*

*# the phase (T60).*

dat <- read.table(~SoleData.csv, header=TRUE, sep=",");

***# Step 2: transform data of raw counts per compartment to proportions per temperature zone***

***# 2a: sum of raw counts over all counting times***

dat$Sum <- rowSums(dat[,c("T0","T10","T20","T30","T40","T50","T60")]);

***# 2b: create a dataset ‘aggdat’ with aggregate counts per experiment, acclimation temperature,***

***# phase and compartment***

aggdat <- aggregate(dat$Sum,list(as.factor(dat$Experiment),as.factor(dat$Acclimatisatie.temp),as.factor(dat$Fase),as.factor(dat$Compartiment)),sum);

***# 2c: create a dataset ‘aggdatsum’ with aggregate counts per experiment, acclimation***

***# temperature and phase***

aggdatsum <- aggregate(dat$Sum,list(as.factor(dat$Experiment),as.factor(dat$Acclimatisatie.temp),as.factor(dat$Fase)),sum);

***# create a dataset ‘expdat’ with each row representing a combination of experiment and phase***

***# (18*3=54 rows) and 8 columns representing the proportions of usage of each compartment***

expdat <- data.frame(aggdatsum,z1=rep(NA,nrow(aggdatsum)),z2=rep(NA,nrow(aggdatsum)),z3=rep(NA,nrow(aggdatsum)),z4=rep(NA,nrow(aggdatsum)),z5=rep(NA,nrow(aggdatsum)),z6=rep(NA,nrow(aggdatsum)),z7=rep(NA,nrow(aggdatsum)),z8=rep(NA,nrow(aggdatsum)));

for ( ii in 1:nrow(aggdatsum) ) {

ttt <- aggdat[aggdat$Group.1==aggdatsum$Group.1[ii] & aggdat$Group.2==aggdatsum$Group.2[ii] & aggdat$Group.3==aggdatsum$Group.3[ii],];

expdat[ii,5:12] <- ttt$x/expdat$x[ii];

};

***# 2d: create data set with proportions per temperature zone, by summing up the proportions of***

***# usage of compartments with the same temperature zone***

*# C1: 28 degrees: compartment 1*

*# C2: 26 degrees: compartments 2 and 8*

*# C3: 23 degrees: compartments 3 and 7# C4: 20 degrees: compartments 4 and 6*

*# C5: 17 degrees: compartment 5*

propdat <- expdat[,1:4];

propdat$C1 <- expdat$z1;

propdat$C2 <- expdat$z2 + expdat$z8;

propdat$C3 <- expdat$z3 + expdat$z7;

propdat$C4 <- expdat$z4 + expdat$z6;

propdat$C5 <- expdat$z5;

names(propdat) <- c("run","treat","ba","x","C1","C2","C3","C4","C5");

***# Step 3: compute log-ratios***

***# take the middle temperature (22 ^o^C) as the denominator***

propdat$LR1 <- log(propdat$C1/propdat$C3);

propdat$LR2 <- log(propdat$C2/propdat$C3);

propdat$LR3 <- log(propdat$C4/propdat$C3);

propdat$LR4 <- log(propdat$C5/propdat$C3);

***# Step 4: offset log-ratios of usage with log-ratios of availability of temperature zones***

propdat$LRA1 <- propdat$LR1 - log(0.5);

propdat$LRA2 <- propdat$LR2 - log(1);

propdat$LRA3 <- propdat$LR3 - log(1);

propdat$LRA4 <- propdat$LR4 - log(0.5);

***# create labels that are more intuitive***

propdat$treatment <- propdat$ba; propdat$treatment <- as.character(propdat$treatment);

propdat$treatment[propdat$treatment == "I"] <- "before";

propdat$treatment[propdat$treatment == "II"] <- "after1";

propdat$treatment[propdat$treatment == "III"] <- "after2";

***# Step 5: assess evidence of non-random usage in the non-gradient phase:***

***# 5a: create a matrix ‘CM’ of log-ratios of the non-gradient phase***

BAdat <- propdat;

BAdat <- BAdat[order(BAdat$treat,BAdat$run,BAdat$ba),]

BALR1 <- BAdat$LRA1[BAdat$ba=="I"];

BALR2 <- BAdat$LRA2[BAdat$ba=="I"];

BALR3 <- BAdat$LRA3[BAdat$ba=="I"];

BALR4 <- BAdat$LRA4[BAdat$ba=="I"];

MYBAdat <- data.frame(run=BAdat$run[BAdat$ba=="I"],treat=BAdat$treat[BAdat$ba=="I"], LRtype=as.factor(c(rep("28/23",length(BALR1)),rep("26/23",length(BALR1)),rep("20/23",length(BALR1)),rep("17/23",length(BALR1)))), LR=c(BALR1,BALR2,BALR3,BALR4));

CM <- rbind(BALR1,BALR2,BALR3,BALR4);

***# 5b: compute the generalized likelihood ratio statistic which compares a model with no***

***# preference against a model with preference (Aebischer, 1993).***

treatseq <- BAdat$treat[!duplicated(BAdat$run)];

H1MAT <- H2MAT <- H3MAT <- matrix(data=NA,nrow=4,ncol=4);

for ( i in 1:4 ) {

for ( j in 1:4 ) {

H1MAT[i,j] <- sum(CM[i,]*CM[j,]);

H2MAT[i,j] <- sum((CM[i,]-mean(CM[i,]))*(CM[j,]-mean(CM[j,])));

};

};

***# test for general preference (deviation from random use)***

TEST <- -ncol(CM)*log(det(H2MAT)/det(H1MAT)); TEST;

1 - pchisq(TEST,df=4);

***# The matrix of log-ratios may also be analysed using MANOVA, for example to assess***

***# evidence of treatment effects:***

fit <- manova(t(CM) ~ as.factor(treatseq)); summary(fit, test="Wilks");
